# Barriers and facilitators to implementing a task-sharing mental health intervention for Sickle Cell Disease populations in low- and middle-income countries: a qualitative analysis using the Consolidated Framework for Implementation Research (CFIR)

**DOI:** 10.3389/fpubh.2025.1607771

**Published:** 2025-07-15

**Authors:** John Patena, Leah Elster, Tania Hameed, Sumedha Kulkarni, Alden Yuanhong Lai, Annika C. Sweetland, Joyce Gyamfi, Temitope Ojo, Angela Odoms-Young, Charmaine Royal, Emmanuel Peprah

**Affiliations:** ^1^Implementing Sustainable Evidence-Based Interventions Through Engagement (ISEE) Lab, Department of Global and Environmental Health, New York University School of Global Public Health, New York, NY, United States; ^2^Department of Public Health Policy and Management, New York University School of Global Public Health, New York, NY, United States; ^3^Columbia University Vagelos College of Physicians and Surgeons/New York State Psychiatric Institute, New York, NY, United States; ^4^Light Institute for Global Health and Transformation, Division of Infectious Diseases, Washington University School of Medicine in St. Louis, St. Louis, MO, United States; ^5^Division of Nutritional Sciences, Cornell University, New York, NY, United States; ^6^Departments of African & African American Studies, Biology, Global Health, and Family Medicine & Community Health, Duke University, Durham, NC, United States

**Keywords:** Sickle Cell Disease, depression, anxiety, task-sharing interventions, LMIC, mental health

## Abstract

**Background:**

People living with Sickle Cell Disease (SCD) experience higher rates of common mental disorders (CMD). There is an alarming treatment gap in the provision of adequate mental health services for CMDs in low- and middle-income countries (LMIC). One solution is the implementation of task-sharing interventions such as the Friendship Bench which utilizes concepts of problem-solving therapy (PST). This investigation uses a qualitative study design to evaluate the acceptability and feasibility of implementing a PST-based task-sharing mental health intervention for SCD populations in LMICs using the Consolidated Framework for Implementation Research (CFIR).

**Methods:**

Purposive, convenience, and snowball sampling strategies were utilized to identify study participants targeting two key groups: (1) SCD stakeholders and (2) global mental health (GMH) experts. Key informant interviews were conducted between July–September 2024. A framework analysis approach was used by iterative deductive and inductive coding. Results were analyzed and synthesized into key themes and patterns, stratified by participant type to highlight variations across stakeholder perspectives.

**Results:**

A total of 16 participants completed key informant interviews: 10 (62.5%) were SCD stakeholders and 6 (37.5%) were GMH experts. The geographic scope of work spans 12 countries, with 9 (75.0%) located in sub-Saharan Africa. Both SCD stakeholders and GMH experts expressed a shared consensus on the urgent need for mental health care tailored to SCD populations in LMIC settings. Implementing a task-sharing mental health intervention was viewed as acceptable, however, perspectives on its feasibility varied. Identified barriers included the absence of robust health care systems, limited prioritization and funding for mental health, a shortage of trained mental health professionals, and the pervasive stigma surrounding both SCD and mental health conditions. Conversely, facilitators included the potential receptiveness of SCD populations to mental health care delivered by task-sharing providers, the integration of mental health services within SCD clinics to avoid external referrals, and the cultural adaptability of PST-based interventions.

**Discussion:**

Challenges associated with implementing task-sharing mental health interventions stem from larger systemic issues within healthcare systems and the integration of care. Task-sharing represents a critical component of the solution, but requires complementary, coordinated efforts to strengthen the health system holistically.

## Introduction

Sickle Cell Disease (SCD) is a genetic blood disorder that leads to several chronic medical complications (1, 2). SCD is a lifelong disease with an overall lower quality of life, higher use of medical resources, and increased economic burden for patients, caregivers, and the healthcare system (1–3). The global burden of SCD is a significant public health issue with high mortality rates among those living in low- and middle-income countries (LMIC) (1, 4, 5). Over 8 million people were living with SCD globally in 2021, with more than 75% of whom residing in countries in sub-Saharan Africa (1). Nigeria reports the largest population affected with SCD and the highest burden of the disease globally (6–8).

Evidence-based interventions for SCD management primarily target SCD treatment goals including relieving symptoms, avoiding pain episodes, and preventing further medical complications (1, 5). Health-related quality of life (HRQoL) is a commonly utilized indicator to measure self-perceived health status, physical health, mental health, and overall function among those living with chronic conditions (9). People living with SCD experience poorer HRQoL overall, with many SCD providers and SCD researchers advocating that interventions for SCD management must improve HRQoL in addition to standard SCD treatment goals (10–12).

Experiencing mental health challenges is common in people living with SCD. The most prevalent psychiatric symptoms are depression and anxiety, collectively referred to as common mental disorders (CMD) (12–18). Depression rates in SCD adults globally report an average prevalence of 24% (as high as 85%) (19) compared to the global average of adults with depression of 5% (20). Additionally, there is strong evidence between pain frequency and higher depressive and anxiety symptoms (21). Social determinants of health that contribute to SCD-related mental health challenges include food and housing insecurity, unequal access to healthcare, and medical discrimination including accusations that patients are seeking recreational drugs when they are actually seeking relief from pain (22). SCD patients also experience stigma from SCD which has a complex relationship to mental health conditions and psychosocial HRQoL (22–24). This stigma is often rooted in misconceptions of SCD (e.g., that the disease is contagious) and cultural beliefs around SCD (e.g., that the disease is a “punishment for ancestral sins”) (22). Additionally, emotional pain (pain resulting from psychological experiences) may be an important component of physical pain (25). These stressors of social determinants of health, stigma, and pain exacerbate the symptoms of CMDs for people living with SCD. This evidence points to the strong need to incorporate mental health support in SCD care and management.

Existing mental health interventions for SCD primarily highlight its use for pain management, with none specifically addressing CMDs and psychosocial HRQoL. Currently, mental health interventions for SCD fall under the categories of cognitive techniques, behavioral change techniques, increasing social support, and patient education, with innovative efforts to introduce digital-delivered (e.g., internet, mobile health) interventions. Cognitive Behavioral Therapy (CBT) is the predominant approach in mental health interventions for SCD, but outcome measures mainly focus on the management of pain (26–32).

There is a significant treatment gap in the provision of adequate mental health services for CMDs in LMICs. Up to 90% of individuals in need of mental health care do not receive it, and amongst those that utilize mental health services do not receive adequate treatment. This gap is driven by a severe shortage of mental health professionals, high cost of and financial barriers to care, and the pervasive stigma associated with seeking help related to mental health issues (33–35). Estimates suggest that LMICs only have 1.4 mental health professionals per 100,000 people, (36, 37) highlighting the urgent need to address this disparity in the treatment of mental health.

One effective solution to closing this treatment gap is the implementation of task-sharing interventions, which aims to expand access to mental health care amongst the most vulnerable populations (38, 39). Task-sharing involves redistributing care traditionally provided by mental health specialists (e.g., psychologists and psychiatrists) to non-specialists, such as community or lay health workers (LHW), through structured training and supervision (40, 41). This strategy has been widely adopted in global mental health initiatives and has proven to be an effective means of increasing mental health service delivery in resource-constrained settings. However, while various task-sharing mental health interventions to address CMDs are well-documented in LMICs, much of the literature emphasizes the need for further scaling and broader implementation of these programs (42–44).

One prominent example of an evidence-based task-sharing intervention, the Friendship Bench, utilizes concepts of problem-solving therapy (PST), a structured, step-by-step approach that empowers individuals to identify their problems and develop workable solutions (45). The Friendship Bench intervention was originally developed in Zimbabwe and has been extensively studied and demonstrated effectiveness in reducing CMDs broadly in other LMICs including Kenya, Malawi, and Tanzania (46–55). Delivered through six sessions by trained LHWs, the intervention uses a manualized script and is implemented within primary care facilities under the supervision of a mental health professional. The full description of the intervention and how it was developed are described elsewhere (51, 56, 57).

Current guidelines for the treatment and management of SCD lack a comprehensive approach to address the mental health needs of people living with this condition. The National Heart, Lung, and Blood Institute (NHLBI) published the *Evidence-Based Management of Sickle Cell Disease: Expert Panel Report, 2014*, (58) the World Health Organization (WHO) African Region *WHO Sickle Cell Disease Package of Interventions*, (59) the Sickle Pan-African Research Consortium (SPARCo) *Standards of Care for Sickle Cell Disease in Sub-Saharan Africa*, (60) the American Society of Hematology *Clinical Practice Guidelines on Sickle Cell Disease*, (61) the European Hematology Association *Hemoglobinopathies Initiatives*, (62) and the British Society for Hematology *Guidelines for the Management of Sickle Cell Disease* (63) provide detailed guidance to standardize and enhance the management of SCD. These guidelines address critical components of SCD care, such as pain management, the prevention of complications, and the use of disease-modifying therapies like hydroxyurea. However, these guidelines lack a clear and comprehensive emphasis on mental health care for people living with SCD.

It is evident that SCD requires further resources dedicated to mental health interventions that specifically target decreasing CMDs and improving psychosocial HRQoL. PST has emerged as a promising psychological modality to address CMD in chronic diseases particularly within LMICs, however, has not been investigated with SCD. Given the proven success of the Friendship Bench intervention in reducing CMDs for various populations in LMICs, there is an opportunity to investigate if this PST-based intervention is a novel approach to addressing the mental health needs of people living with SCD.

This investigation uses a qualitative study design to evaluate the acceptability (degree to which the intervention is perceived as appropriate and/or satisfactory) and feasibility (degree to which the intervention is practical and can be implemented effectively within the context of the available resources and constraints) of a PST-based, task-sharing mental health intervention tailored for people living with SCD in LMICs. By exploring these dimensions, this investigation aims to inform the potential for adapting and scaling this intervention to improve mental health outcomes for SCD populations in resource-limited settings.

## Methods

### Setting

Key informant interviews drew from a larger NHLBI funded study “mAnaging siCkle CELl disEase through incReased AdopTion of hydroxyurEa in Nigeria” (ACCELERATE), an implementation trial with an embedded clinical trial which examines the adoption of hydroxyurea as an evidence-based intervention to manage SCD in Nigeria. The study is a partnership between New York University (NYU) School of Global Public Health and the Center of Excellence for Sickle Cell Research and Training at the University of Abuja (CESRTA) in Abuja, Nigeria. CESRTA is the central administrative hub of SCD providers and researchers from 25 healthcare centers that comprise the Sickle Pan African Research Consortium NigEria Network (SPARC-NEt). In turn, SPARC-NEt is part of SPARCo, a larger consortium comprised of 7 countries—Ghana, Mali, Nigeria, Tanzania, Uganda, Zambia, and Zimbabwe—whose mission is to develop research capacity for SCD across Africa (64). Ethics approvals for the key informant interviews were obtained as part of the larger ACCELERATE study by the Institutional Review Boards of NYU Langone Health and the University of Abuja Teaching Hospital, and the Nigerian National Health Research Ethics Committee.

### Sampling and recruitment

Purposive sampling strategy was used to identify study participants who were either: (1) SCD stakeholders (e.g., experts in SCD care/management, SCD clinicians/providers, SCD researchers), or (2) global mental health (GMH) experts (e.g., experts in developing, implementing, or scaling up task-sharing mental health interventions in LMICs, mental health clinicians/providers, mental health researchers). Convenience sampling was used from contacts within ACCELERATE, SPARC-NEt, SPARCo, and other professional contacts of authors (JP, AS, EP). Researchers created an initial list of SCD stakeholders and GMH experts to contact. Participants were recruited via email. Snowball sampling was also used as some participants referred other potential participants within their respective professional networks. Recruitment was conducted until thematic saturation was assessed by the lead author (JP).

### Study design and approach

The qualitative study utilized the Consolidated Framework on Implementation Research (CFIR), a widely recognized implementation science determinant framework that can be used to understand, guide, and evaluate the factors that influence the successful implementation of interventions, treatments, or policies (65). CFIR is the most commonly cited framework to assess implementation of single interventions (66). The framework was revised in response to recommendations aimed at enhancing its applicability across diverse settings (referred to as CFIR 2.0) (67). CFIR 2.0 consists of 39 constructs organized into 5 key domains: (1) Innovation, (2) Process, (3) Individuals, (4) Inner Setting, and (5) Outer Setting. These domains assess the multifaceted elements that influence the uptake/adoption and sustainability of interventions. In this study, CFIR provided a structured framework to analyze and interpret the findings from the key informant interviews, specifically in relation to the implementation of PST-based task-sharing mental health interventions.

A semi-structured interview guide was developed based on CFIR concepts utilizing the CFIR Interview Guide Tool (68) and with input from experts in qualitative study design (AL), SCD research (EP), and GMH research (AS). Key informant interviews were conducted between July 2024 to September 2024 via the ZOOM videoconference platform by the lead author (JP) who has extensive experience in qualitative research methods. To mitigate potential interviewer bias, JP utilized the semi-structured interview guide and conducted interviews with active listening and reflective. Interviews were audio recorded using a handheld recorder, transcribed verbatim by NYU Stream Transcription Service, and de-identified. Interview transcripts were uploaded to Dedoose qualitative software (Version 9.0.17, SocioCultural Research Consultants LLC, Los Angeles, California, 2021). Before the interview, participants completed a brief demographic survey via REDCap (69) which captured anonymized information on areas of expertise, professional roles, educational background, geographic scope of their work, and gender.

### Analysis

A framework analysis approach was used to qualitatively analyze the key informant interviews (70). Framework analysis is an iterative process that involves both deductive (theory-driven) and inductive (data that emerges) coding. The process included:

**Step 1. Familiarization**: Initial transcripts were reviewed to identify key topics that were emerging from the interviews.
**Step 2. Analytic framework:** The qualitative analysis codebook was deductively developed using CFIR domains/constructs as parent codes and sub-codes. Transcripts were then coded with an inductive approach to find patterns. Codes were added, removed, and combined as needed. A final version based on the CFIR framework was developed and iteratively adapted for the content of the key informant interviews.
**Step 3. Indexing:** The codebook was uploaded to Dedoose to help organize codes and aid in the process of coding. Codes were assigned to relevant excerpts in the transcripts. The main author (JP) coded all transcripts, and 3 co-authors (LE, TH, SK) served as second coders dividing the transcripts among themselves. The second coders met with JP individually every 2 transcripts to ensure concordance and to discuss any discrepancies in coding. Final decisions on coding were based on consensus.
**Step 4. Charting:** Coded excerpts were systematically organized within the developed analytic framework to compare and identify patterns of barriers and facilitators to implementation across the various CFIR domains.
**Step 5. Interpretation:** The chartered data were analyzed for key themes and patterns, stratified by responses from SCD stakeholders and GMH experts.

## Results

### Participant characteristics

A total of 16 participants completed key informant interviews: 10 (62.5%) were SCD stakeholders and 6 (37.5%) were GMH experts. The average length of interviews was 42 min (range: 15–53 min). A majority identified as female (68.7%). All had postgraduate degrees as their highest educational background, and many held multiple degrees (e.g., medical and master's degrees). Among SCD stakeholders, most provide SCD care/management as SCD clinicians/providers and conduct SCD research, with only 2 in SCD policy/advocacy. All GMH experts provide mental health care and conduct mental health research, with 4 also in mental health policy/advocacy. One of the GMH experts has specific expertise in providing mental health care to SCD populations. The remainder provide general mental health care.

Both SCD stakeholders and GMH experts reported additional expertise in public health interventions either in developing, implementing, or evaluating programs (37.5%), conducting public health research (31.3%), or in public health policy/advocacy (25.0%). Moreover, 10 participants conduct their work in a global health context (62.5%). The average years of experience in their respective areas of expertise is 18.43 (standard deviation: 7.85). Most participants identified as being a researcher (81.3%) and/or a healthcare provider (68.7%), with fewer as a public health professional (37.5%), educator (12.5%), policymaker (6.3%), or community health worker/community advocate (6.3%). A majority work in a university or educational institution (68.7%) and/or a healthcare facility or health system (62.5%), with fewer in a non-governmental agency (18.8%), community-based organization (18.8%), non-profit organization (12.5%), mental health facility (12.5%), or governmental agency (6.3%).

**Figure 1:**
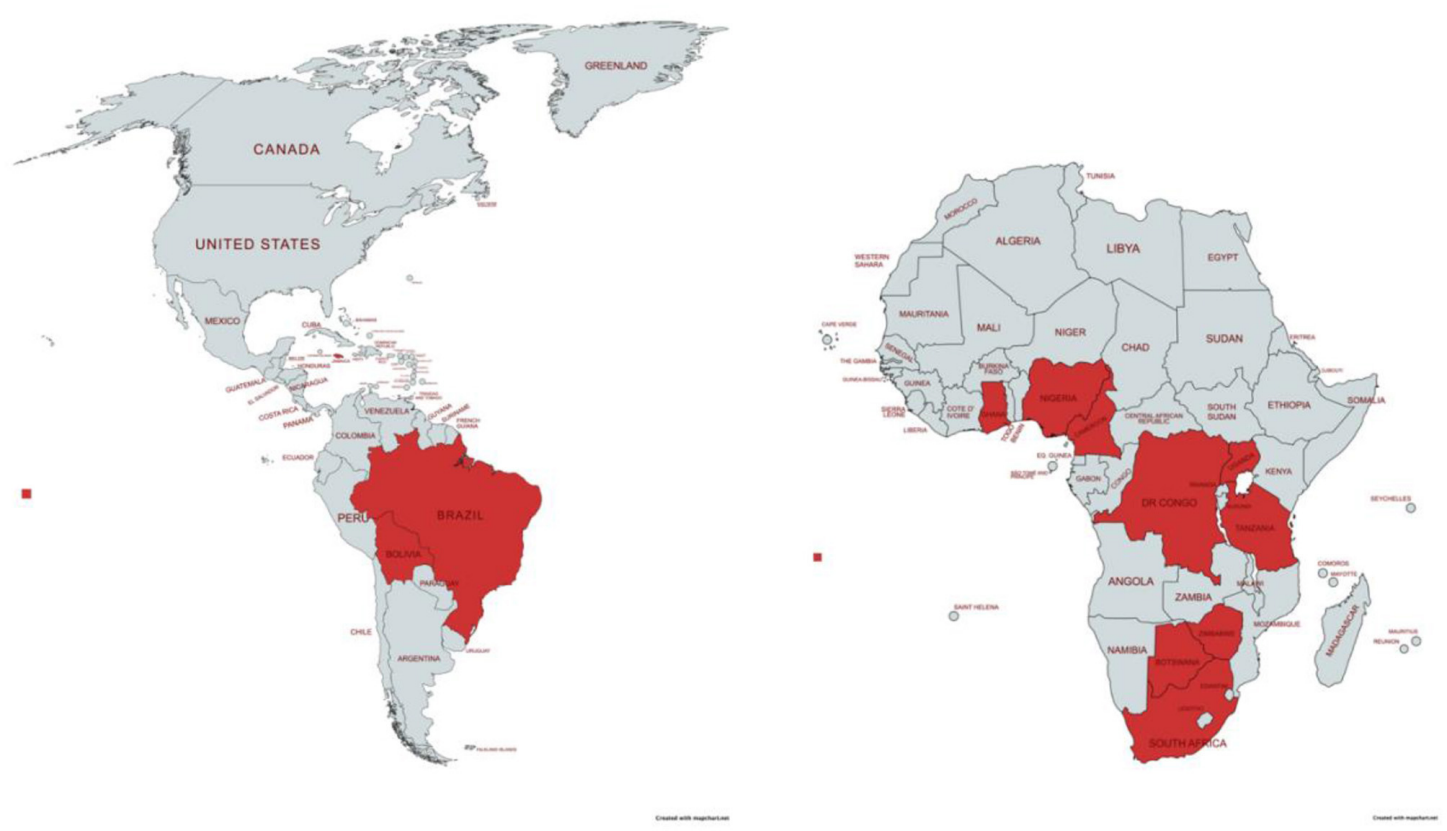
Map of geographic scope of work.

The geographic scope of work for all participants spans 12 countries in total, with 9 countries (75.0%) located in sub-Saharan Africa: Botswana, Cameroon, Democratic Republic of Congo, Ghana, Nigeria, South Africa, Tanzania, Uganda, and Zimbabwe. The remaining countries include Bolivia, Brazil, and Jamaica (see Figure 1). The country with the most representation of scope of work was Jamaica (31.3%), followed by Nigeria (25.0%). Some participants reported conducting specific work in these 12 countries as well as a general scope of work at the regional level including Africa (25.0%), Asia (12.5%), the Caribbean (25.0%), Latin America (12.5%), and globally (i.e., all regions) (6.3%). All participant characteristics are found in Table 1.

**Table 1:** Key informant participant characteristics.

**Participant characteristics**	***N* = 16 (%)**
**Key informant type**	
SCD stakeholder	10 (62.5)
GMH expert	6 (37.5)
**Sex**	
Female	11 (68.7)
Male	5 (31.3)
**Educational background** ^*^	
Medical (e.g., MD, MBBS, MBChB)	11 (68.7)
Doctorate (e.g., PhD)	6 (37.5)
Masters (e.g., MPH, MSW, MSc, MA)	9 (56.3)
**Areas of expertise** ^*^	
Global health	10 (62.5)
SCD (clinical)	9 (56.3)
SCD (research)	9 (56.3)
SCD (policy/advocacy)	2 (12.5)
GMH (clinical)	6 (37.5)
GMH (research)	6 (37.5)
GMH (policy/advocacy)	5 (31.3)
Public health interventions (develop/implement/evaluate)	6 (37.5)
Public health interventions (research)	5 (31.3)
Public health (policy/advocacy)	4 (25.0)
Average years of expertise experience	18.43 (SD: 7.85)^+^
**Professional roles** ^*^	
Researcher	13 (81.3)
Healthcare provider	11 (68.7)
Public health professional	6 (37.5)
Mental health provider	4 (25.0)
Educator	3 (12.5)
Policymaker	1 (6.3)
Community health worker/community advocate	1 (6.3)
**Work setting** ^*^	
University or educational institution	11 (68.7)
Healthcare facility or health system	10 (62.5)
Non-governmental agency	3 (18.8)
Community-based organization	3 (18.8)
Non-profit organization	2 (12.5)
Mental health facility	2 (12.5)
Governmental agency	1 (6.3)
**Geographic scope of work** ^*^	
Bolivia	1 (6.3)
Botswana	1 (6.3)
Brazil	1 (6.3)
Cameroon	1 (6.3)
Democratic Republic of Congo	2 (12.5)
Ghana	1 (6.3)
Jamaica	5 (31.3)
Nigeria	4 (25.0)
South Africa	1 (6.3)
Tanzania	2 (12.5)
Uganda	1 (6.3)
Zimbabwe	2 (12.5)

^*^Exceeds 100% as participants can select multiple options.

^+^SD, Standard Deviation.

### Qualitative themes

There was broad consensus among SCD stakeholders and GMH experts regarding the urgent need for mental health care tailored to SCD populations. While task-sharing mental health interventions were generally regarded as highly acceptable to address this need, perspectives on its feasibility were mixed (see Table 2).

**Table 2:** CFIR barriers and facilitators by participant type.

**CFIR domains and constructs**	**SCD stakeholders**		**GMH experts**	
	**Barriers**	**Facilitators**	**Barriers**	**Facilitators**
**Innovation domain:** ***The evidence-based intervention being implemented***.				
**Innovation relative advantage** *The innovation is better than current practice or other available innovations*.	–	-Provides support for mental health that SCD care would not usually focus on	-Perceived as insufficient compared to more comprehensive services and care	-Provides support that could be well received and be helpful
**Innovation adaptability** *The innovation can be modified, tailored, or refined to fit local context*.	-Referring out for mental health care, need to keep within SCD clinic	-Leverage existing virtual networks that SCD communities have created	-Uncertainty of where to deliver the intervention to ensure privacy	-Need to collaborate with local stakeholders and understand dual stigma with SCD
**Process domain:** ***The activities and strategies used to implement the innovation***.				
**Assessing needs** *Collect information about priorities, preferences, and needs of the target group*.	-Confusing pain management needs with only physical symptoms	-Keep assessment within SCD clinics, not send out for psychiatric referral	–	-LHWs are able to assess low intensity needs and serious mental illness
**Training** *The process of training or providing education to implement the innovation*.	-Training currently focuses or prioritizes the main health condition	-Training and education for both providers and patients about SCD and mental health	-Already asking LHWs to do so much, adding mental health might be too much	-Training needs to be comprehensive for sustainability
**Individuals domain:** ***The roles and characteristics of individuals involved***.				
**Role of implementer** *Individuals who lead or execute efforts to implement the innovation*.	-Current SCD providers do not feel comfortable assessing for mental health	-Knowledgeable and skilled enough	–	-Identifying with a LHW is more receptive -LHW acts as a liaison between points of care
**Inner setting domain:** ***The setting in which the innovation is implemented***.				
**Structural characteristics** *The physical infrastructure or workflows within the inner setting*.	-Lack of available mental health services -Priority for SCD conditions, without time for mental health	-Meet patients where they are at to provide local care	-Lack of available mental health services in clinics -Overburden of mental health workforce	–
**Outer Setting:** ***The larger context in which the inner setting (and innovation) exists***.				
**Policies and laws** *Legislation, guidelines, or recommendations that support implementation*.	-Lack of SCD clinical guidelines and funding for mental health	–	-Lack of a strong health system overall	-Integrating mental health services into primary health care
**Local attitudes** *Sociocultural values and beliefs that support implementation*.	-Dual stigma of SCD and mental health	–	–Misconceptions of what mental illness is	–

**Innovation domain:** This domain encompasses the evidence-based intervention that is being implemented, which in this context, is the Friendship Bench. The construct *Innovation Relative Advantage* assesses how the innovation proposed performs compared to existing/current practice or other alternative innovations. Both participant types reported facilitators related to the perceived effectiveness of task-sharing interventions. Specifically, SCD stakeholders emphasized that such an intervention could provide crucial mental health support that is currently lacking in SCD practices which typically do not prioritize mental health concerns:

“*I do think that interventions that really provide for the person who is participating in the intervention to have more immediate kind of feedback like cognitive behavioral therapy or problem-solving therapy or motivational interviewing. I think that that is, I strongly support that and think that it has sort of the best bang for your buck.” -SCD stakeholder*

GMH experts highlight that task-sharing mental health interventions could be well received and effective among SCD populations:

“*I would say it's really been received positively. […] And I think it also may be the group work approach in Africa. Most of the things that people do in Africa and the communities, the group work approach has always been the best approach to really address things in the African communities. Because people believe in the spirit of togetherness and the spirit of unity, and they believe it's not an individual problem once something affects the person, it affects the group. Group approach has been an easier approach to a compared to an individual approach in the African setting.” -GMH expert*

Barriers in this construct were reported by GMH experts that there could be a perception from patients that task-sharing mental health interventions do not provide sufficient solutions. They describe how those seeking help often look for medications, and that perhaps a task-sharing mental health intervention may not be “enough”:

“*People's minds are quite fixed on medical models. They feel that a professional, what they do, is prescribe drugs. Patients often feel validated by having a prescription as well. And they might feel that, you know, someone talking to me doesn't really address how bad I feel. You know, it's not a serious enough solution to the problems I have.” -GMH expert*

The construct *Innovation Adaptability* refers to perceptions about how the Friendship Bench can be modified, tailored, or refined to fit local contexts. SCD stakeholders reported that task-sharing mental health interventions can leverage the existing virtual networks that communities have created. Using the messaging communication platform WhatsApp, SCD communities have formed social support groups where they already share their experiences with SCD:

“*I think would be also very good because our patients are very active in the digital space […] where people could use their phones because our patients use their phones for everything. To log their pain, reach out to relatives. They have, you know, support groups there. So I think for our patients, you know, if the Friendship Bench was a virtual space, that would be something a lot of our patients would benefit from” -SCD stakeholder*

GMH experts highlighted the need to collaborate with local stakeholders to understand the dual stigma within mental health and SCD. Doing so will provide great insights that will prove invaluable for tailoring the intervention to the local population and context:

“*I feel like in a co-creation session with this intervention is on the clinicians and the stakeholders involved on the ground. It would be to actually assess this and look at the pros and cons. And you know Sickle Cell also has the stigma in Nigeria as well. So you're looking at two conditions that are very massively stigmatized and trying to get them to create something that will work and wouldn't be affected by either of the perception of that condition. It's going to be very difficult to do, but also it doesn't mean it's not possible, right?” -GMH expert*

Barriers for this construct include SCD stakeholders' concerns of needing to refer out for mental health care:

“*It's just to where to put it. I think for all patients, if we had a psychologist provided within our clinic space, that would be something that would really benefit our patients” -SCD stakeholder*

GMH experts echoed this sentiment, and brought up the uncertainty of where to deliver the intervention to ensure patient privacy:

“*we're really having to be very delicate about if we put this in a non-clinical setting […] How do you also protect those who might be seen going to the intervention or using it, and at what cost, right?” -GMH expert*

**Process domain:** This domain describes the activities and strategies used to implement the innovation. The construct *Assessing Needs* reports on collecting information about the priorities, preferences, and needs of the target group. Facilitators from SCD stakeholders included keeping the assessment of mental health needs within SCD clinics, without the need for an external psychiatric referral:

“*a quick assessment and saying, okay, you have, you know, clinical depression, mild or moderate or anxiety or so and you need further assessment. […]Would you be okay with maybe talking to this person and you can just come right back here and, you know, I'll give you a date and you can just sit and talk with this person for 20 min and see if, you know, this test that we just did, if it's the start of a little red flag that we need to address early or so it would be easier for us to like, you know, say, ok, here is everything kind of handed to you. All you have to do is show vs. sending them to a different facility.” -SCD stakeholder*

GMH experts emphasized that task-sharing providers are able to assess patients with low intensity psychological needs vs. those who need more support for serious mental illness:

“*You don't want to miss a patient who is endorsing suicidal ideation. You don't want to miss a patient who is floridly psychotic. Are you worried you know, is this a patient who is depressed? Is this a patient who is anxious? Do they have depression and anxiety? They probably have both getting that exact diagnosis correct for four sessions of talk therapy? Does it really matter? Probably not. Again, this is for low intensity interventions. So for me, the fork in the road is, is this a patient with severe mental illness? Is this not? And I think if a lay counselor can make that distinction, that is a skill that would be paramount.” -GMH expert*

Barriers for the process of implementing the Friendship Bench were brought up by SCD stakeholders highlighting that SCD populations may be confusing pain management needs with only physical symptoms and not addressing the psychological impacts of pain:

“*Even with pain being like the most frequent complication of Sickle Cell Disease, persons patients included they kind of because it's something that they're physically feeling. They don't consider the mental aspect of that that pain management as well. So instead of addressing it, they tend to lean more on pharmaceuticals, which in and of itself can cause other complications or worsen other complications” -SCD stakeholder*

The construct *Training* describes the process of training or providing education to implement the innovation. Facilitators include that training must be provided for both providers and patients/families about SCD and mental health, and that the trainings need to be comprehensive to establish sustainability:

“*Everyone talks about task sharing, right? That's the obvious, and I'm sure that will be a dominant theme that will come across in your research. And there are good models of task sharing that work. There's strong evidence for task sharing. With the caveat, we can't overburden the existing workforce. We need to be mindful of quality implementation. Training needs to be rigorous. We need supervision mechanisms, we need proper renumeration. Right. All of those human resources for health parameters that need to be in place for appropriate task sharing. But it is a model that can work if implemented smartly and resources are available to support those who are doing the shared task.” -GMH expert*

Barriers for the implementation process include the fact that LHWs (and other non-mental health specialists) are already asked to do so much in task-sharing. Training is currently focused on the medical or chronic health condition, not prioritizing mental health care:

“*workload of generalists of both at primary level, also community health workers. Everyone is thinking about trying to get them to do their thing as well. You have dermatologist trying to teach front community health workers, basic dermatology. You've got the eye people wanting to make sure that every front line health worker can do a basic eye test. You know, people are trying to do basic physio for children with the cerebral palsy. There is a real challenge and there's a lot pushback now from community workers saying, there is only so much we can do” -GMH expert*

**Individual domain:** This domain describes the roles and characteristics of individuals involved in the implementation process. The construct *Role of Implementer* specifically covers individuals who lead or execute efforts to implement the innovation. Facilitators from SCD stakeholders' perspectives highlight that implementers must be knowledgeable and skilled enough to make decisions of when to triage:

“*so the person would have to be trained to kind of when they call they call they can identify, okay, this one, we need to send somebody to the house to do a wellness check. You know, this person needs to be brought straight to the hospital. This person, we can refer to a psychologist that can do some intense counseling, or this person just needs to talk.” -SCD stakeholder*

GMH experts' stated that LHWs are more approachable as they are seen as trusted members of the community, and patients would be more receptive to their support. Additionally, LHWs can serve as liaisons between different points of care for a patient (e.g., doctor, nurse, family member):

“*I think the whole idea of Friendship Bench as a concept, in that it might actually be very effective for Sickle Cell, because of the idea of having someone who's older, who feels like a mother, who feels like a grandmother. They're more receptive. They understand pain, they've been through a lot. So it might be that they can be more open to that kind of intervention, might be something that might be effective.” -GMH expert*

Barriers in this construct highlight that SCD providers do not feel comfortable conducting mental health assessments themselves:

“*I can use the depression screening tool and so on. But I am not comfortable, you know, not as comfortable treating something that's not as simple […]But once it becomes something a little bit more than that, I feel like I feel the need to get more specialist care or to, you know, refer and say, Hey, I have patients, and I refer, you know, I'd rather you treat this person than me. So I think I guess there if there are ways to I guess make it more, you know, structured, like you said, you create guidelines that we could be used to I guess make us more comfortable.” -SCD stakeholder*

**Inner setting domain:** This domain describes the setting in which the innovation is implemented, which in this context, includes healthcare settings and clinics. The construct *Structural Characteristics* describes the physical infrastructure or clinical/organizational workflows within the implementation context. Facilitators described by SCD stakeholders included that SCD clinics need to meet patients where they are at:

“*It's always like having to do, you know, not too much medical jargon, you just have to bring it to their level, like incorporate local dialects, you know, make it engaging. A little bit entertaining, sometimes to really capture their attention that's typically how people are. Otherwise, they're just kind of also depends on who delivers the message as well.” -SCD stakeholder*

Barriers in the implementation context include GMH experts' acknowledgment that mental health care systems are lacking in most LMIC settings, impacting the available services that can be provided in local health clinics. Additionally, there is a lack of available clinics that can provide mental health support because of the overburden of the workforce and shortage of mental health specialists:

“*You have a breakdown in communication because you don't have that continuum of care. Partly because the system is overburdened, you have health workforce shortages. You don't have health information technology that allows for the communication that you need between providers you still have a lot of out of pocket payment, which means that the patient can't complete the care cascade because they don't have the resources to follow through the care seeking behavior. So, in addition to the fragmentation, you have a disruption of the care. So yes, fragmentation and disruption of care.” -GMH expert*

An SCD stakeholder echoes the same sentiment of a lack of available resources for clinics to provide sufficient mental health care:

“*A few local NGOs among the foundation of the current First Lady provide some level of psychological, clinical, and social support, but these resources are often limited and are not sufficient to meet the needs of all patients. Kinshasa has a population of close to 15 million inhabitants.” -SCD stakeholder*

Additionally, SCD stakeholders pointed out that SCD patients primarily come in for SCD support, without thinking about mental health needs. SCD clinics prioritize SCD care, not because of a lack of importance for mental health, but because there are such limited resources:

“*A lot of people might only come when they're ill, and that's definitely not the time. You know, somebody comes in and severe painful crisis. You're just so busy dealing with that that, you know, there's a group of patients who will only come when they're ill unfortunately even though they'll always be given appointments. So, we're probably not diagnosing enough and we're not referring enough.” -SCD stakeholder*

**Outer setting domain:** This domain describes the larger context in which the inner setting (and innovation) exists. The *Policies & Law* construct encompasses legislation, guidelines, or recommendations that support implementation, including the role of governments and health systems. GMH experts reported that the field at large is supportive of a decentralized healthcare system through the integration of mental health support at the primary care level:

“*one of our big advocacy messages within our health systems work is about decentralization, particularly in low and middle income countries. It's about decentralizing traditional tertiary care mental health services delivered at psychiatric institutions and tertiary hospitals and pushing that care to primary care and community care levels” -GMH expert*

Additionally, GMH experts were hopeful for external funding sources to support the sustainability of mental health care:

“*So many low-income concepts actually, is never going to be magic. You're always going to have a like fightback intentional or intentionally by the political system. We're not interested in investing in that kind of model. You need external funding to kick this off. You need a standard funding to pilot it, and then when you get the external funding, you also need to make sure the government is involved in even developing this initial phase so that they know that this funding says in four years, the government needs to take ownership of this model and take it forward.” -GMH expert*

However, GMH experts also acknowledge that in order to do this, the larger health system needs to be stronger. Currently, most health systems, especially in LMICs, still lack the necessary overarching infrastructure:

“*The major issue for integrating into primary care is where you have generally an extremely weak health system. So if most of the primary care in the country don't have running water or electricity, and they're falling down, very difficult to bring a high quality mental health service into an environment where the general state of infrastructure is incredibly poor, you know, if the nurses don't get paid from month to month, not very motivated to add another concurrent for health care.” -GMH expert*

SCD providers focused on the fact that SCD clinical guidelines lack the guidance needed for mental health care:

“*we have clinical care guidelines too, but, you know, like I wouldn't say that mental health issues is a very big chunk of that guideline. In terms of being as structured as, like, all the issues are as structured, you know, where it has, like, you know, step by step ways to approach it. I wouldn't say that it is as structured.” -SCD stakeholder*

Additionally, SCD providers highlighted that national policies and support from governments significantly lack mental health funding:

“*So definitely the budget constraints and in terms of what the government allocates to mental health services. So also, I guess probably I don't I'm not even sure what the numbers are in terms of, like, trained medical health professionals and stuff? In the population, but I mean, I know that it definitely is under served in terms of how many people actually need it. So I guess shortage of the professionals specialize for that area as well.” -SCD stakeholder*

The construct *Local Attitudes* largely encompasses the sociocultural values and beliefs that support implementation. Across both participant types, stigma around mental health and SCD were deeply rooted in sociocultural beliefs, serving as major barriers to implementing mental health care. Among SCD stakeholders, mental health was still perceived as a condition for “mad people”:

“*everyone knows once you hear about [psychiatric ward], that the person going there is mad. So considering that most of their clinics occur at the ward, then trying to convince the patients to take a litter to go to that ward, I yeah. It's a little bit of a challenge in and of itself. Some of the health centers also have mental health clinics on special days of the weeks. But of course, that is also noted by persons in the general community. Oh, if she going to clinic on such and such date, that means that she going to the mad people. You have that kind of negative statement that you're kind of trying to overcome, trying to help your patients.” -SCD stakeholder*

SCD stakeholders also discussed the dual stigma that exists with living with SCD and a mental health condition:

“*There is definitely some stigma and discrimination, I think you did mention that. You know, just culturally, you know, in our setting, you know, any mental health issues is kind of frowned upon and I think it comes from, you know, lack of public awareness and, you know, probably there needs to be more robust campaigning in terms of bringing more awareness to it to reduce the amount of stigma that's associated. I think that some patients probably don't seek as much care as more people probably do need to seek care because of fear of being stigmatized” -SCD stakeholder*

GMH experts specifically commented on the overall stigma of mental health, bringing to light the complexity and often times misconceptions of mental illness:

“*Stigma contributed to the mental health gap anywhere in the country and anywhere in the world, but also in the country. Yeah. Stigma is a big component. Yeah. And then to have a national mental health stigma campaign is one of the is one of the key deliverables. Yeah. So yeah, and then people are present for mental conditions. You know, you have psychotic disorders, but the truth is in most of these chronic medical conditions, is not so much of psychosis, but common mental disorders like depression and anxiety.” -GMH expert*

## Discussion

Both SCD stakeholders and GMH experts recognized the significant need and demand for mental health services for SCD populations. Each group's perspective did not contradict one other but instead focused on different aspects of acceptability and feasibility that were more salient to them. SCD stakeholders' responses skewed more toward individual characteristics, innovation characteristics, and processes that impacted the SCD population. GMH experts' responses skewed more toward macro-level characteristics such as the mental health workforce, health system strengthening, and the overall theme of integrating mental health care with chronic disease care. These perspectives add a well-rounded view of the complex topic of increasing mental health care for this population. With both perspectives, we can begin to investigate further how to tackle the issue from all angles.

Task-sharing mental health interventions are widely acknowledged and effectively utilized to address CMDs in LMICs (47). Adapting PST-based interventions for SCD populations was viewed as highly acceptable, however, feedback regarding their feasibility is mixed. It is noteworthy to observe that the challenges identified are not unique to SCD but common across many task-sharing mental health interventions. These challenges reflect broader systemic issues in health systems and the integration of care, which are essential for meeting mental health needs for individuals with chronic care conditions including SCD. Other considerations are what aspects of the Friendship Bench are most important for this population? Is it the physical bench? Is it the curriculum delivered by LHWs? These answers will help tailor task-sharing mental health interventions specific to SCD populations.

A recurring theme from the interviews was the understanding that task-sharing mental health interventions alone cannot bridge the larger mental health treatment gap. The shortage of mental health professionals in most communities is a pressing issue. Health system strengthening is crucial, particularly in expanding both the primary health care and mental health workforce, while also promoting the integration of mental health care into broader health services.

Another overarching theme was the need for a robust mental health system to which patients can be referred for more intensive support. For individuals requiring more comprehensive mental health services, communities currently lack an adequate health infrastructure. A key part of the challenge is that LHWs and other task-sharing providers are already overburdened with existing responsibilities. While task-sharing has been successful in addressing several treatment gaps across the public health system, it does not resolve the fundamental issue of an overall shortage of health/medical professionals.

Mental health is often not perceived as the priority—by both providers and patients/caregiver. In LMICs, the chronic *medical* condition (in this case SCD) is always the priority. One SCD provider described, while there is a desire for a comprehensive mental health program within the SCD clinic, the focus remains solely on SCD care due to resource constraints:

“*you can't have a champagne system on a Pepsi budget. I mean, you know, just a resource constrained situation. So, we have to do the best that we can.” -SCD stakeholder*

In this context, the term “champagne system” refers to an ideal, robust mental health program that includes regular screening and adequate number of mental health professionals. In contrast, a “Pepsi budget” symbolizes the reality of limited resources, necessitating the prioritization of SCD care. This stark contrast underscores the broader challenges within the healthcare system, as well as the specific challenges in implementing mental health interventions. Focusing on one area (e.g., training LHWs for task-sharing mental health interventions), inevitably means that other areas will require additional support.

Finally, stigma presents a significant and multifaceted barrier for successful implementation. There is stigma both around living with SCD and with experiencing mental health conditions, and these two intersect, compounding the challenges of help-seeking, adherence to treatment, and overall improvement in quality of life.

Findings from the interviews on improving mental health care is consistent with WHO advocacy for a collaborative care model, which integrates mental health treatment into primary care settings. In September 2025, the UN will host the “Fourth High-Level Meeting on Noncommunicable Diseases and Mental Health” aimed at discussing, defining, and committing to actionable national and policy recommendations that promote the integration of NCDs and mental health within national health financing systems (71, 72). While the focus of the meeting will be on NCDs such as cardiovascular diseases, diabetes, cancer, and chronic respiratory diseases, it may also pave the way for the inclusion of other NCDs, such as SCD. If global movements are mobilized, they could help address several of the barriers identified in the key informant interviews ultimately improving SCD care by integrating mental health services and advocating for comprehensive care. SCD care would improve for millions of people globally by incorporating mental health care and advocacy.

These findings highlight that the conversation of mental health within SCD management is important but not addressed enough. From these stakeholder perspectives, implementing a PST-based task-sharing mental health intervention, such as the Friendship Bench, could be a place to start. Next steps in this implementation research are to understand the perspectives of people living with SCD, and their caregivers/social support systems. By getting these perspectives, we can begin to see a full picture of what is acceptable and feasible to enhance mental health care for the SCD population.

### Strengths and limitations

This qualitative study design guided by the CFIR framework provides valuable insights by capturing perspectives from a broad sector of stakeholders, including representation from several African countries for where SCD is highly prevalent. By gathering perspectives from both SCD stakeholders and GMH experts, this study offers a balanced view of how successful implementation of a mental health intervention for SCD could occur in resource constrained settings. However, this study is subject to some limitations. Convenience sampling introduces selection bias, which limits the generalizability of the findings. Additionally, there was an over-representation of stakeholders from Jamaica, as snowball sampling proved more successful from initial participants from this region. Another limitation is the exclusive focus on stakeholders and experts as participants. While the perspectives of SCD patients and caregivers are crucial, the scope of this investigation was limited to stakeholders or those in positions of broad influence. Future investigations should replicate the process to include the patient and caregiver perspectives.

## Conclusion

This qualitative study assessed the acceptability and feasibility of implementing the Friendship Bench, a PST-based task-sharing mental health intervention for individuals living with SCD in LMICs. Through key informant interviews, the study explored the perceptions of SCD stakeholders and GMH experts. Overall, there was a strong consensus on the pressing need for enhanced mental health care for SCD populations. The findings highlight that by training LHWs in task-sharing interventions like the Friendship Bench, integrating mental health services within SCD clinics, and addressing broader health system barriers to enable integrated care, there is a significant opportunity to adapt and implement this intervention to increase mental health care access for SCD patients in LMICs.

## Data Availability

The raw data supporting the conclusions of this article will be made available by the authors, without undue reservation.
